# Physical Activity, Quality of Life, and Pelvic Floor Disorders Before and After Hysterectomy for Gynecological Cancer: A Prospective Cohort Study

**DOI:** 10.1007/s00192-025-06157-3

**Published:** 2025-05-31

**Authors:** Robyn Brennen, Kuan Yin Lin, Linda Denehy, Sze-Ee Soh, Thomas Jobling, Orla M. McNally, Simon Hyde, Helena Frawley

**Affiliations:** 1 Department of Physiotherapy, The University of Melbourne, Parkville, Vic 3010 Australia; 2 Department of Physiotherapy, The University of South Australia, Adelaide, SA 5000 Australia; 3 Vital Core Physiotherapy, Stepney, SA Australia; 4 School and Graduate Institute of Physical Therapy, College of Medicine, National Taiwan University, Taipei, Taiwan; 5 School of Health Sciences, The University of Melbourne, Parkville, Vic 3010 Australia; 6 The Peter MacCallum Cancer Centre, Melbourne, Vic 3000 Australia; 7 School of Primary and Allied Health Care, Monash University, Frankston, Vic 3199 Australia; 8 School of Public Health and Preventive Medicine, Monash University, Melbourne, Vic 3004 Australia; 9 Monash Health, Moorabbin, Melbourne, Australia; 10 The Royal Women’s Hospital, Parkville, Vic 3010 Australia; 11 The University of Melbourne, Parkville, Vic 3010 Australia; 12 Mercy Hospital for Women, Heidelberg, Vic 3084 Australia

**Keywords:** Pelvic floor disorders, Urinary incontinence, Fecal incontinence, Physical activity, Gynecological cancer

## Abstract

**Introduction and hypothesis:**

This study investigated physical activity (PA) levels and health-related quality-of-life (HRQoL) before and after, and associations between pelvic floor disorders (PFD) and PA/HRQoL after, hysterectomy for gynecological cancer.

**Methods:**

Secondary analysis of a longitudinal cohort study, with assessments before, 6 weeks, and 3 months after hysterectomy for gynecological cancer. The International Physical Activity Questionnaire-short form was used to assess PA and European Organisation for Research and Treatment of Cancer Quality-of-Life Questionnaire-C30 (QLQ-C30) to assess HRQoL. Changes over time were analyzed using generalized estimating equations or linear mixed models. Associations between PFD with PA and HRQoL were analyzed using logistic regressions and analyses of variance.

**Results:**

Of 126 participants, median age 63 years, most had endometrial (69%) and/or stage 1 cancer (72%) and underwent total hysterectomy (65%). Pre-surgery, 39% met PA guidelines, increasing to 53% 3 months post-surgery (+14%, 95% CI 2–25). Mean global health status/QoL domain scores from the QLQ-C30 did not change significantly (+4.4/100 points, 95% CI −0.8 to 9.6). Three months post-surgery, whether participants met PA guidelines was not associated with PFD. Participants with anal incontinence or double incontinence had lower global health status/QoL scores than participants without these symptoms (mean (SD) AI 61.4 (17.8) vs no AI 72.5 (18.7), *p* = 0.006; DI 61.5 (17.9) vs no DI 71.7 (18.8), *p* = 0.019).

**Conclusion:**

PA levels were low pre- and post-surgery but worse preoperatively. This presents an opportunity for prehabilitation interventions to optimize physical function. There was no association between meeting PA guidelines and PFD. Global health status/QoL was slightly below population norms pre- and post-surgery. Lower HRQoL was associated with some symptoms of PFD.

**Supplementary Information:**

The online version contains supplementary material available at 10.1007/s00192-025-06157-3.

## Introduction

Gynecological cancers are among the most common types of cancer in women, comprising 12% of all new cancer cases diagnosed in women in the United States of America in 2024 [1], and 9% of all new cancer cases diagnosed in women in Australia in 2024 [2]. The most common of these gynecological cancers are cervical, endometrial (including uterine sarcomas), and ovarian cancer [1], with rates of endometrial and ovarian cancer expected to increase into the future [1]. Gynecological cancer treatments include surgery, radiotherapy, and chemotherapy, with hysterectomy+/-oophorectomy being the most common treatment for gynecological cancer. These treatments may contribute to pelvic floor disorders, including urinary incontinence, fecal incontinence and dyspareunia [3]. The prevalence of pelvic floor disorders has been found to be higher after gynecological cancer treatment than in the general population [4].

Physical activity is recommended during and after cancer treatment [5], with physical and mental health benefits demonstrated for women after gynecological cancer treatment [6, 7]. Prehabilitation, programs targeted at improving physical fitness and psychological well-being after diagnosis and before surgery is recommended for patients with gynecological cancer, to improve their health prior to surgery and enhance recovery [8]. Despite these recommendations, low physical activity levels post-surgery have been identified in patients undergoing surgery for gynecological cancer [9, 10]. Furthermore, several studies have identified associations between pelvic floor disorders and low levels of physical activity in individuals without cancer [11, 12], and increased symptoms of pelvic floor disorders have been associated with lower levels of moderate-to-vigorous physical activity in women with advanced ovarian cancer [13]. However, associations between pelvic floor disorders and physical activity have not been investigated in women with other gynecological cancers, or those who have had hysterectomy for gynecological cancer. Associations between pelvic floor disorders and physical activity in women who have hysterectomy for gynecological cancer need to be investigated in longitudinal cohort studies, as this would support research into whether treating pelvic floor disorders can improve patient general health and well-being after surgery.

In addition to compromised physical well-being, gynecological cancer treatment is an emotionally and socially challenging experience for many patients. Cancer diagnosis and side-effects of cancer treatment have been associated with low HRQoL and emotional function for women diagnosed with gynecological cancer [3]. However, existing studies have not examined changes in HRQoL during the early recovery period after hysterectomy for gynecological cancer [3]. Qualitative research indicates that pelvic floor disorders cause considerable distress and have a negative impact on HRQoL after gynecological cancer treatment [3, 14]. Gynecological cancer survivors with urinary and/or fecal incontinence report reduced HRQoL and restricted participation in activities of daily living and social activity [14]. There are inconsistent results across cross-sectional studies that have investigated associations between urinary incontinence and HRQoL after gynecological cancer treatment [15, 16], and no studies were found investigating associations between fecal incontinence and HRQoL in this population. If symptoms of pelvic floor disorders are shown to be associated with lower HRQoL after hysterectomy for gynecological cancer, it would be important to explore interventions that may improve the HRQoL and experience of gynecological cancer survivors in this early recovery period.

### Objectives

This is a secondary analysis of a cohort study that examined the prevalence and severity of pelvic floor disorders in women before and after hysterectomy for gynecological cancer and has been reported in a previous publication. In this publication we report on the secondary aims, which were to identify changes in physical activity levels and HRQoL from before to after hysterectomy for gynecological cancer and determine associations between pelvic floor disorders and whether participants met physical activity guidelines, and between pelvic floor disorders and HRQoL, after hysterectomy for gynecological cancer. We hypothesized that fewer participants would meet physical activity guidelines and HRQoL would be lower at 3 months post-surgery and that participants with pelvic floor disorders would be less likely to meet physical activity guidelines and have lower HRQoL compared to participants who did not report pelvic floor disorders.

### Methods

This observational study is reported according to the STrengthening the Reporting of OBservational studies in Epidemiology (STROBE) statement [17]. Ethics approval was obtained from the Monash Health Human Research Ethics Committee (NMA HREC Reference Number: HREC/44667/MonH-2018-151163(v)). The longitudinal cohort study was a level II prognostic study [18] that collected data on pelvic floor disorders, physical activity levels and HRQoL (outcomes of interest) across time before and after the exposure to hysterectomy for gynecological cancer. As such, no comparator group was required.

### Setting

This study was conducted in gynecology–oncology outpatient clinics in public hospitals and private clinics in Melbourne, Australia. Participants underwent hysterectomy for gynecological cancer between July 2019 and August 2022.

### Eligibility Criteria

We included women (≥18 years old) who were scheduled for hysterectomy for endometrial/uterine, ovarian or cervical cancer. Exclusion criteria were pregnancy, severe neurological disorders, severe physical or psychiatric impairments, or inability to communicate in English.

### Recruitment

Electronic patient lists from the gynecology–oncology clinics at participating hospitals in metropolitan Melbourne, were screened by a member of the research team to identify potentially eligible patients. Potentially eligible participants were identified prior to surgery once the surgical plan for a hysterectomy was confirmed, or in the first week after hysterectomy. These patients were screened initially by clinical staff for inclusion and exclusion criteria and asked for consent to be contacted by a researcher. The researcher contacted patients in person or by phone to discuss study details and provide consent forms (paper-based or online). Patients who consented were given baseline questionnaires (paper-based or online). Eligible patients who returned consent forms and completed baseline questionnaires within 7-days after surgery were included.

### Outcomes

#### Sociodemographic, Medical, and Pelvic Floor Disorder Data

Sociodemographic, medical, and pelvic floor disorder data were collected as previously reported in Brennen et al. [19]. In brief, sociodemographic and medical data collected at baseline included age, height, weight, parity, home situation, relationship status, education level, employment status, smoking status, medical history, cancer history, and cancer treatment. Data on symptoms of pelvic floor disorders collected at baseline, 6 weeks after surgery, and 3 months after surgery included the prevalence of urinary incontinence, moderate-to-severe urinary incontinence (score >2 on the Incontinence Severity Index), fecal incontinence, anal incontinence (flatal and/or fecal incontinence), double incontinence (presence of both urinary and anal incontinence), sexual activity, and dyspareunia. The prevalence and distress of symptoms in the domains of overall urogenital symptoms, pelvic organ prolapse symptoms, and colorectal-anal symptoms, as well as overall pelvic floor dysfunction were assessed using the Pelvic Floor Distress Inventory (PFDI-20) [20] and were also collected at baseline, 6 weeks after surgery, and 3 months after surgery.

#### Physical Activity and HRQoL Data

Physical activity was assessed at baseline, 6 weeks after surgery, and 3 months after surgery. For participants recruited pre-surgery, the International Physical Activity Questionnaire (IPAQ-7), which has been shown to have good concurrent validity [21], was used at all timepoints. For participants recruited post-surgery, baseline physical activity was assessed using questions on the number of hours per week undertaken for vigorous and moderate physical activity and walking prior to surgery, with no specified timeframe, and post-surgical physical activity levels were assessed using the IPAQ-7. Responses were assessed to determine whether these met the US Department of Health and Human Services physical activity guidelines of at least 150 min of moderate intensity, or at least 75 min of vigorous intensity, aerobic physical activity per week [22].

Health-related quality-of-life was assessed using the European Organisation for Research and Treatment of Cancer Quality-of-Life Questionnaire (QLQ-C30 version 1.0 (QLQ-C30(V1))), which has been designed and validated to assess HRQoL in oncology populations, over a 1-week period of time [23]. The QLQ-C30 incorporates a global health status/QoL scale, five functional scales (physical, role, cognitive, emotional, and social), three symptom scales (fatigue, pain, and nausea), and a number of single items assessing additional symptoms commonly reported by cancer patients (dyspnea, loss of appetite, insomnia, constipation, and diarrhea) and perceived financial impact of the disease. Participants recruited post-surgery were asked to rate their pre-surgery health and quality-of-life using the QLQ-C30 global health status/QoL domain questions, with no specified timeframe. For the global health status/QoL and functional scales, higher scores indicate greater HRQoL or greater function. For the symptom domains/items higher scores indicate greater severity or negative impact of symptoms.

### Sample Size

The sample size calculation, aiming to recruit 125 participants, was calculated for the primary aims of the longitudinal study [19], based on the primary outcome measure of any urinary incontinence on the Incontinence Severity Index. This planned secondary analysis is an exploratory analysis.

### Statistical Methods

Baseline outcomes data and change in physical activity and HRQoL outcomes over time were visually assessed for normality. The proportion of participants that met physical activity guidelines was reported for each timepoint. The distribution of the QLQ-C30 domains scores were highly skewed at each timepoint; therefore, median and interquartile ranges were used to describe the data at each time point.

The changes between timepoints in whether participants met the guidelines for physical activity (a binary outcome) were assessed using generalized estimating equations (GEEs) using a binomial outcome distribution with participants as the subject effect variable and timepoint as the within subject effect variable. The changes between timepoints in QLQ-C30 physical function, emotional function, and global health status/QoL domain scores were assessed using linear mixed models (LMMs) with participants as a random effect and timepoint as a categorical fixed effect. Participants were included in the longitudinal analyses if they provided data for the outcome being analyzed for at least one follow-up timepoint. Missing data were not imputed due to the small sample size and exploratory nature of the study [24].

To determine the impact of recruiting a proportion of participants post-surgery, sensitivity analysis was conducted. Analysis of the whole cohort and analysis of participants recruited pre-surgery only was conducted for the proportion of participants who met physical activity guidelines, the QLQ-C30 global health status/QoL domain and changes in these two outcomes over time.

The associations between the presence of specific pelvic floor symptoms (urinary incontinence, fecal incontinence, and scores of >0 on the PFDI-20 and the domains of the PFDI-20) and whether participants met physical activity guidelines at 3 months post-surgery were assessed using single variable logistic regression models. Associations between the presence of each pelvic floor symptom and global health status/QoL at 3 months post-surgery were assessed using one-way analysis of variance (ANOVA). For analyses of outcomes at 3 months post-surgery, participants were excluded if they had not provided data for the outcome being analyzed.

All statistical analyses were conducted using IBM SPSS (version 27) with *p* values set at 0.05.

## Results

Participant flow is shown in Supplementary Fig. 1. Of 126 participants, 107 completed baseline questionnaires between cancer diagnosis and surgery and 19 completed baseline questionnaires retrospectively in the first week after surgery. Follow-up questionnaires were provided by 113 (90%) participants 6 weeks following surgery, and 110 (87%) participants at the 3-month follow-up timepoint.

### Participant Characteristics

Demographic and cancer treatment details are presented in Table 1. Further characteristics have been previously reported in Brennen et al. [19]. In summary, the median age was 63 years (range 30–99), most were post-menopausal (94/126 75%), 69% had endometrial cancer, and 62% had stage 1 cancer. Thirty-five percent of participants had a radical hysterectomy, 33% had an open surgical approach, and 48% received adjuvant or neo-adjuvant therapy as well as surgery.

**Table 1:** Participant demographic and cancer treatment characteristics

Characteristic	*n* = 126
Age median (IQR)	63 (15)
BMI median (IQR)	32.1 (10.2)
**All values* n *(%)**	
Stage of cancer	
Stage 1	78 (62)
Stage 2	18 (14)
Stage 3	19 (15)
Stage 4	11 (9)
Location of primary cancer	
Endometrium or uterus	92 (73)
Ovaries	28 (22)
Cervix	6 (5)
Type of hysterectomy	
Radical	44/126 (35)
Non-radical	82/126 (65)
Surgical approach	
Open	42/126 (33)
Laparoscopic	84/126 (67)
Concomitant salpingo-oophrectomy	
BSO	117/126 (93)
USO	3/126 (2)
Concomitant pelvic lymph node dissection	55 (44)
Adjuvant therapy	
Surgery only	63 (50)
Surgery + adjuvant therapy	61 (48)
Surgery + chemotherapy	− 25 (20)
Surgery + radiotherapy	− 21 (17)
Surgery + chemotherapy + radiotherapy	− 11 (9)
Adjuvant therapy type unknown	− 4 (3)
Unknown whether had adjuvant therapy	2 (2)

*IQR* Inter quartile range; *BMI* body mass index

#### Summary of Physical Activity and HRQoL Outcomes at Each Timepoint

A summary of the physical activity, HRQoL, and pelvic floor outcomes at each timepoint is shown in Table 2. Over a third of participants (39%) met physical activity guidelines at baseline, decreasing to 31% 6 weeks after surgery and increasing to more than half of the participants (whole cohort 53%, participants recruited pre-surgery only 54%) by 3 months after surgery. Median global health status/QoL and physical function scores remained the same (67/100 and 87/100, respectively) at all timepoints. Median emotional function scores increased from 75/100 before surgery to 92/100 at 6 weeks and 3 months after surgery.

**Table 2:** Summary of physical activity, HRQoL, and pelvic floor outcomes at each timepoint

Outcome	Time 1: baseline (pre-surgery)	Time 2: 6 weeks post-surgery	Time 3: 3 months post-surgery
**All values** ***n *(%)**			
International Physical Activity Questionnaire/Physical activity questions			
Physical activity guidelines met	46/117 (39)	31/101 (31)	54/102 (53)
Physical activity guidelines met (sensitivity analysis excluding participants recruited after surgery)	38/98 (39)	25/82 (31)	47/87 (54)
Incontinence Severity Index			
Any urinary incontinence	83/126 (66)	60/112 (54)	65/110 (59)
Moderate-to-very severe urinary incontinence	41/125 (33)	34/110 (31)	37/110 (34)
Pelvic Floor Distress Inventory			
Anal incontinence	27/126 (21)	34/112 (30)	31/110 (28)
Faecal incontinence	15/126 (12)	10/112 (9)	15/110 (14)
Double incontinence			
Double incontinence	21/126 (17)	23/112 (21)	25/110 (23)
Pelvic Floor Distress Inventory			
PFDI-20 > 0	107/126 (85)	89/110 (81)	78/110 (71)
POPDI-8 > 0	67/126 (53)	58/112 (52)	51/110 (46)
CRADI-8 > 0	76/126 (60)	66/112 (59)	59/110 (54)
UDI-6 > 0	94/126 (75)	67/112 (60)	60/110 (55)
Female Sexual Function Index/Sexual function questions			
Sexually active	34/126 (27)	29/113 (26)	46/110 (42)
Dyspareunia	11/17 (65)	3/8 (38)	11/20 (55)
**All values Median (IQR)**			
European Organisation for Research and Treatment of Cancer Quality of Life questionnaire#			
Global health scale (higher scores indicate higher level of function)			
Global health status/QoL	67 (33)	67 (25)	67 (25)
Global health status/QoL (sensitivity analysis excluding participants recruited after surgery)	67 (33)	67 (25)	67 (25)
Functional scales (higher scores indicate higher level of function)			
Physical function	87 (33)	87 (27)	87 (27)
Role function	100 (33)	67 (50)	87 (27)
Cognitive function	83 (33)	83 (33)	83 (33)
Emotional function	75 (25)	92 (33)	92 (33)
Social function	83 (33)	83 (33)	100 (33)
Symptom scales/items (higher scores indicate more symptoms and greater negative impact)			
Fatigue	33 (44)	33 (33)	0 (33)
Pain	17 (50)	17 (33)	33 (33)
Nausea	0 (17)	0 (0)	0 (0)
Dyspnea	0 (33)	0 (17)	0 (33)
Appetite	0 (33)	0 (33)	33 (33)
Insomnia	33 (67)	33 (67)	0 (33)
Constipation	0 (33)	0 (33)	0 (0)
Diarrhea	0 (0)	0 (0)	0 (33)
Financial impact	0 (33)	0 (0)	0 (0)

*QoL* quality of life; *IQR* interquartile range

# Number of responses at each timepoint for the European Organisation for Research and Treatment of Cancer Quality of Life questionnaire domains/items are as follows:

Global health status/QoL: T1 *n* = 125, T2 *n* = 113; T3 *n* = 107

Global health status/QoLsensitivity analysis

T1 *n* = 106; T2 = 94; T3 = 90

Physical function: T1 *n* = 102, T2 *n* = 113, T2 *n* = 108

Role function, Social function, Fatigue, Pain, Nausea, Dyspnea, Diarrhea, Financial impact:

T1 *n* = 99, T2 *n* = 113; T3 *n* = 108

Cognitive function: T1 *n* = 99; T2 *n* = 112; T3 *n* = 108

Emotional function: T1 *n* = 96; T2 *n* = 113; T3 *n* = 108

Appetite, Constipation: T1 *n* = 98, T2 *n* = 112; T3 *n* = 108

Insomnia: T1 *n* = 99, T2 *n* = 113, T3 *n* = 107

#### Changes in Physical Activity and HRQoL Over time

The results from the GEEs and LMMs are shown in Table 3. At 3 months after surgery compared to before surgery, there were statistically significant increases in the proportion of participants who met physical activity guidelines (whole cohort: difference in proportions 14%; 95% CI 2, 25, participants recruited pre-surgery only: difference in proportions 15%; 95% CI 3, 28) and statistically significant improvements in median scores in the emotional function domain of the QLQ-C30 (MD 10.0; 95% CI 3.4, 16.6). There were no statistically significant changes in the global health status/QoL or physical function domains of the QLQ-C30.

**Table 3:** Changes in physical activity and HRQoL outcomes over time

Outcome	Pairwise comparisons T2–T1	Pairwise comparisons T3–T2	Pairwise comparisons T3–T1
**All values difference in proportions [95% CI]**			
International Physical Activity Questionnaire/Physical activity questions			
Physical activity guidelines met	−9 [−20, 3]	22 [10, 34]*	14 [2, 25]*
Physical activity guidelines met (sensitivity analysis excluding participants recruited post-surgery)	−8 [−22, 5]	24 [10, 37]*	15 [3, 28]*
**All values mean difference [95% CI]**			
European Organisation for Research and Treatment of Cancer Quality of Life questionnaire			
Global health status/QoL	3.9 [−1.2, 9.1]	0.5 [−4.7, 5.6]	4.4 [−0.8, 9.6]
Global health status/QoL (sensitivity analysis excluding participants recruited post-surgery)	4.7 [−0.9, 10.3]	0.2 [−5.6, 6.0]	4.9 [−0.8, 10.5]
Physical function	−1.3 [−6.9, 3.1]	2.4 [−3.0, 7.7]	1.0 [−4.8, 6.8]
Emotional function	8.2 [1.6, 14.7]*	1.9 [−4.6, 8.3]	10.0 [3.4, 16.6]*

*statistically significant

#### Associations Between Pelvic Floor Disorders and Physical Activity, Pelvic Floor Disorders, and HRQoL

At 3 months post-surgery, there were no significant associations between the presence or absence of symptoms on any of the pelvic floor disorders and whether participants met physical activity guidelines (Table 4.).

**Table 4.**
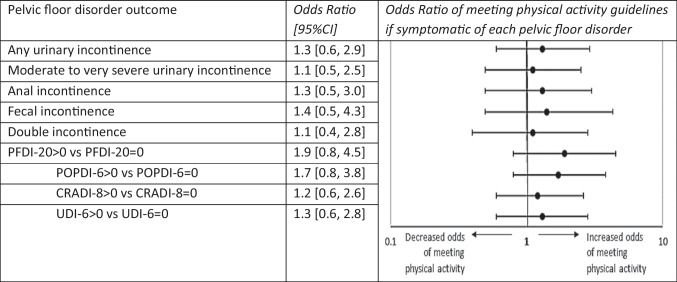
Odds ratios of meeting physical activity guidelines in the presence of specific pelvic floor disorder outcomes

*CRADI-8*, Colorectal Anal Distress Inventory-short form; *PFDI-20*, Pelvic Floor Distress Inventory-short form; *POPD-8*, Pelvic Organ Prolapse Distress Inventory-short form; *UDI-6*, Urogenital Distress Inventory-short form

As shown in Table 5, participants with anal incontinence had lower mean global health status/QoL scores (mean 61.4, SD 17.8) than participants who did not have anal incontinence (mean 72.5, SD 18.7, *p* = 0.006), and participants with double incontinence had lower mean global health status/QoL scores (mean 61.5, SD 17.9) than participants who did not have double incontinence (mean 71.7. SD 18.8, *p* = 0.019). Participants who reported pelvic floor symptoms on the PFDI-20, pelvic organ prolapse symptoms on the POPDI-8 subdomain, and colorectal-anal symptoms on the CRADI-8, at 3 months post-surgery, had lower global health status/QoL scores than participants who did not report symptoms on these outcome measures. Global health status/QoL scores were not significantly different between participants who reported symptoms and those who did not report symptoms on other pelvic floor outcome measures.

**Table 5:** Univariate analyses of pelvic floor disorders and HRQoL

Pelvic floor disorder outcome	QLQ-C30 global health status/QoL domain score mean ± SD	*p* value
Any urinary incontinence vs	68.4 ± 18.6	0.49
No urinary incontinence	70.9 ± 19.8	
Moderate to very severe urinary incontinence vs	69.1 ± 17.0	0.91
No or slight urinary incontinence	69.6 ± 20.1	
Anal incontinence vs No anal incontinence	61.4 ± 17.8 72.5 ± 18.7	0.006*
Fecal incontinence vs	62.2 ± 13.3	0.12
No fecal incontinence	70.6 ± 19.6	
Double incontinence vs No double incontinence	61.5 ± 17.9 71.7 ± 18.8	0.019*
PFDI-20 > 0 vs	66.7 ± 18.4	0.02*
PFDI-20 = 0	76.0 ± 19.1	
POPDI-6 > 0 vs	61.0 ± 17.8	<0.001*
POPDI-6 = 0	76.6 ± 17.1	
CRADI-8 > 0 vs	64.1 ± 18.5	0.001*
CRADI-8 = 0	75.5 ± 17.8	
UDI-6 > 0 vs	67.5 ± 17.6	0.25
UDI-6 = 0	71.8 ± 20.5	
Sexually active vs	69.1 ± 17.9	0.88
Sexually inactive	69.7 ± 19.9	
Penetrative intercourse attempted vs	75.8 ± 16.0	0.10
No penetrative intercourse attempted	68.0 ± 19.4	

*CRADI-8* colorectal anal distress inventory short form; *OR* odds ratio; *PFDI-20* pelvic floor distress index short form; *POPDI-6* pelvic organ prolapse distress inventory short form; *UDI-6* urogenital distress inventory short form

*statistically significant

## Discussion

The proportion of participants meeting physical activity guidelines and the median scores on the emotional function domain of the QLQ-C30 increased from below equivalent population norms before hysterectomy to well above equivalent population norms by 3 months after hysterectomy, while scores on the QLQ-C30 global health status/QoL domain remained consistent, slightly below equivalent population norms [25]. Symptoms of pelvic floor disorders were not associated with whether participants met physical activity guidelines. Some pelvic floor outcomes, specifically anal incontinence and double incontinence were associated with lower global health status/QoL scores 3 months post-surgery.

The higher proportion of participants meeting physical activity guidelines at 3 months post-surgery suggests that participants were more active after surgery compared to the period between diagnosis and surgery. This contrasts with previous literature that found lower physical activity levels post-surgery compared to before surgery [9, 10]. However, two of the studies included in the systematic review by Lin and colleagues assessed baseline physical activity levels before cancer diagnosis [9]. One of the reasons we observed an increase in physical activity levels post-surgery could be related to the low levels of physical activity observed at baseline in our study. Low baseline physical activity in our cohort may relate to the timing of the baseline outcome measures, as participants were asked about their physical activity levels between diagnosis and surgery. The period of time between diagnosis and surgery is a unique period, in which patients emotionally process their diagnosis [3], while also managing the logistics associated with attending appointments and preparing for surgery. It may be that physical activity decreases after diagnosis compared to before diagnosis and this difference in the baseline period may account for the difference between our results and previous studies [9]. If possible, future studies should examine physical activity levels before diagnosis, between diagnosis and treatment, and after treatment in both the short-term and long-term, to assess whether diagnosis and subsequent treatment impact physical activity levels differently. However, it would require very large population-based studies to prospectively capture pre-diagnosis data and is beyond the scope of most studies. Low levels of physical activity between diagnosis and surgery may indicate an opportunity for intervention, in line with recommendations for prehabilitation for patients diagnosed with gynecological cancer, recognizing that this may depend on multiple factors, including the timing of diagnosis and the interval between diagnosis and surgery [8]. The feasibility of interventions to increase physical activity prior to surgery should be investigated with the goal of thereby reducing post-surgical recovery time and morbidity, and improving post-surgical HRQoL [8].

There were no statistically significant associations between pelvic floor disorders and meeting physical activity guidelines for participants in our study. Although symptoms of pelvic floor disorders have been shown to be a barrier to exercise in non-cancer populations [26, 27], non-pelvic floor-related barriers and enablers to physical activity may be more influential during early recovery after surgery for gynecological cancer. As identified in previous literature other factors such as mood [28], pain, and fatigue [29] may be more influential on physical activity during this early recovery phase. The baseline median emotional function score (75/100) of our participants were below Australian population norms for women (79/100) and for Australians of similar age (60–69 years 82/100) [25] and improved to well above (92/100) these population norms after surgery. The improved emotional function demonstrated by our participants after surgery may have influenced the proportion of participants meeting physical activity guidelines; however, we did not statistically test for associations between emotional function and physical activity levels. It is also possible that participants in our study who had lower symptom levels of pelvic floor disorders when inactive after surgery, subsequently engaged in more physical and social activity as their recovery progressed, which increased their exposure to triggers for pelvic floor disorders. Therefore, possible associations between pelvic floor disorders and physical activity levels early after surgery may have been masked by inactivity and easy toilet access.

Baseline global health status/QoL scores (67/100) were slightly below Australian population norms for women (70/100) and for Australians of similar age (60–69 years 71/100) [25]. Mean global health status/QoL improved post-surgery; however, this was not statistically significant and the median global health status/QoL scores remained consistent. This may relate to our early (6 weeks to 3 months) post-surgery assessment timeframes, in which participants were still recovering from surgery and many were having adjuvant therapy. In previous studies, statistically significant improvements in HRQoL have been found 6 months post-surgery [30] and 12 months, but not 3 months, post-radiotherapy [31]. These results indicate a need for longer-term follow-up as HRQoL may continue to improve after the early post-surgery timepoint that we evaluated.

In our study, participants reporting symptoms of pelvic floor disorders had lower HRQoL scores at 3 months post-surgery, and this was significant for anal incontinence, double incontinence, and the presence of symptoms on the PFDI-20, PFDI-20 colorectal-anal subdomain and PFDI-20 pelvic-organ prolapse subdomain. This indicates that pelvic floor disorders may have a negative impact on HRQoL after gynecological cancer surgery. Previous literature has found associations between urinary incontinence and low HRQoL in non-cancer populations [32]; however, there is limited research examining associations between pelvic floor disorders and HRQoL after gynecological cancer treatment. Larger longitudinal studies are warranted to further evaluate these associations, especially to assess for potential associations between the development of pelvic floor disorders and changes in HRQoL. Clinicians could also consider screening and offering treatment for these symptoms in the early post-surgical recovery period, with the hopes that improving them may potentially improve HRQoL.

### Limitations

This is an analysis of secondary outcomes from an observational study into pelvic floor disorders after gynecological cancer and was exploratory in nature. As only a small proportion of participants developed *de novo* incontinence, statistical analysis of these sub-groups was not conducted to test associations between *de novo* incontinence and the outcomes of interest, limiting the ability of this study to establish causality regarding the potential influence of pelvic floor disorders on physical activity levels or HRQoL. There was heterogeneity in the treatments received by patients, including variations in type of hysterectomy (total or radical), type of surgical approach (open or minimally invasive), and whether patients had neo-adjuvant or adjuvant radiotherapy and/or chemotherapy as well as surgery. Larger studies with greater numbers of participants would allow for greater precision, and better comparison between patients with different stages of cancer, types of hysterectomy, surgical approaches or types of adjuvant therapy. For the minority of participants who completed their baseline questionnaires after surgery, responses about pre-surgery physical activity and HRQoL may have been subject to recall bias. To address this, baseline questionnaires were completed within 7-days after surgery, minimizing the potential time for recall bias and sensitivity analyses were conducted for these outcomes, which demonstrated similar results regardless of whether participants recruited post-surgery were included in the analyses. Statistical significance was not adjusted for multiple comparisons and this should be taken into account when interpreting results. Missing data were not imputed due to the size and exploratory nature of this study, which may have led to biased results particularly for the GEEs. Further larger studies are needed to address these limitations and test hypotheses generated from our results, such as that physical activity may increase post-surgery compared to between diagnosis and surgery, that post-surgical physical activity levels may relate to emotional function, and that some pelvic floor disorders may be associated with lower HRQoL.

Despite these limitations, strengths of this study were the longitudinal nature of the study and the assessment of pre-surgery outcomes either prior to surgery or with only a short delay. We were able to assess physical activity levels and HRQoL before and in the 3 months after hysterectomy for gynecological cancer, providing unique insight into how these outcomes change over this period of time. This also allowed us to identify potential opportunities for increased support or interventions to improve these outcomes at different times in the gynecological cancer journey, particularly between cancer diagnosis and surgery.

## Conclusions and Future Directions

Physical activity levels were low before and after surgery but worse prior to surgery. Emotional function scores were slightly lower than population norms before surgery and substantially higher after surgery. Global health status/QoL scores were slightly lower than population norms before and after surgery, with no significant change. Low physical activity levels and low emotional function between diagnosis and surgery may indicate an opportunity for prehabilitation to optimize patient health and emotional well-being in the time between gynecological cancer diagnosis and treatment. This may depend on factors including the timing of diagnosis and surgery, and future research should investigate the barriers, enablers, and feasibility of prehabilitation in this population. Post-surgery, whether physical activity guidelines were met did not appear related to pelvic floor disorders. Three months after hysterectomy, anal incontinence and double incontinence were associated with lower general health status/QoL. Clinicians could consider screening and offering treatment for these symptoms specifically.

## Supplementary Information

Below is the link to the electronic supplementary material.

Supplementary file1 (PNG 34 KB)

## Data Availability

Raw data are not publicly available in order to protect patient privacy.
