# Supplementary figures and images for: Physical Activity, Quality of Life, and Pelvic Floor Disorders Before and After Hysterectomy for Gynecological Cancer: A Prospective Cohort Study
Source: Int Urogynecol J. 2025 May 31;36(10):2073–83. doi: 10.1007/s00192-025-06157-3 (PMC12618281; doi:10.1007/s00192-025-06157-3)

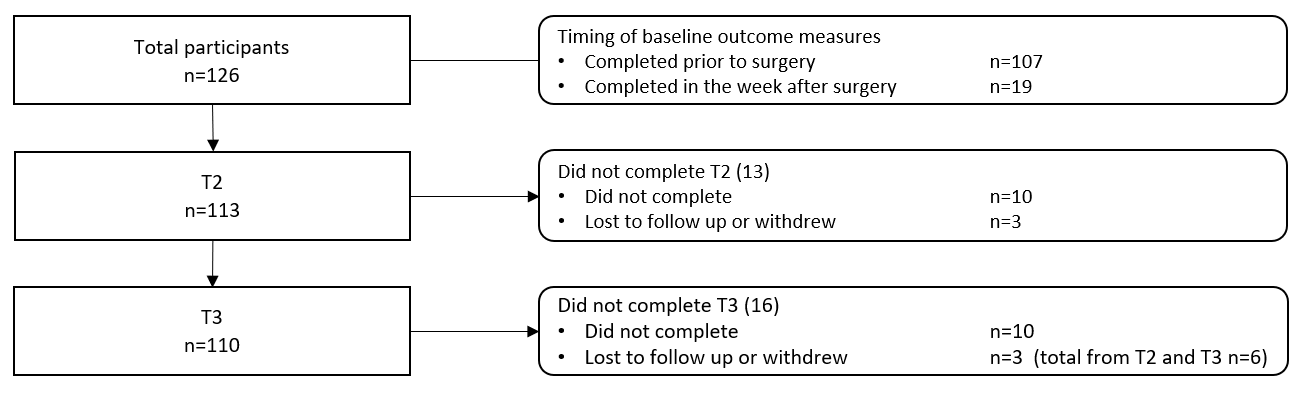

Supplement: Supplementary file 1 — Supplementary file1 (PNG 34 KB) [file 192_2025_6157_MOESM1_ESM.png]
